# Teamspezifische Auswirkungen der Corona-Pandemie auf Mitarbeiter:innen der Internistischen Intensivmedizin eines Krankenhauses der Maximalversorgung

**DOI:** 10.1007/s00063-022-00959-9

**Published:** 2022-09-19

**Authors:** Jochen Wolff, Jürgen Becker, Jan-Hendrik Naendrup, Jorge Garcia Borrega, Jan-Michel Heger, Laura Hamacher, Boris Böll, Dennis A. Eichenauer, Alexander Shimabukuro-Vornhagen, Matthias Kochanek

**Affiliations:** 1 First Department of Internal Medicine, Faculty of Medicine and University Hospital Cologne, University of Cologne; Center for Integrated Oncology Aachen Bonn Cologne Dusseldorf (CIO), Kerpener Str. 62, 50937 Köln, Deutschland; 2 Seelsorge Uniklinik Köln, Universitätsklinik Köln, Köln, Deutschland

**Keywords:** SARS-CoV-2-Infektion, Intensivmedizin, Mortalität, Stresserfahrung, Psychologische Belastung, Wahrnehmung, SARS-CoV‑2 infection, Critical care, Mortality, Stress experience, Psychological stress, Perception

## Abstract

Die anhaltende Belastung der Mitarbeiter im Gesundheitsdienst während der COVID-19-Pandemie ist erheblich und stellt die Mitarbeiter vor große emotionale und psychologische Herausforderungen. In einer teaminternen Evaluation (Ärzt:innen und Pflegekräfte) wurden teamspezifische Belastungen, mögliche Entlastungsstrategien, positive und negative Erfahrungen sowie Wünsche für eine Verbesserung der Situation auf einer Intensivstation erhoben. Während beide Berufsgruppen gleich hohe emotionale Belastungsintensitäten wahrnahmen, werden bei der Pflege zusätzlich starke Belastungsintensitäten im organisatorischen und körperlichen Bereich wahrgenommen. Damit erweist sich die Berufsgruppe der Pflegenden als am stärksten durch die COVID-19-Pandemie belastet. Durch die hier herausgearbeiteten Erkenntnisse können für die Zukunft konkrete Handlungsanweisungen abgeleitet werden.

## Abstract (en)

The ongoing strain on personnel in the healthcare system during the COVID-19 pandemic is considerable and poses major emotional and psychological challenges for the personnel. In a team evaluation (physicians and nurses), team-specific stress, possible relief strategies, positive and negative experiences, and wishes for improvement of the situation in an intensive care unit were collected. While both occupational groups perceived equally high emotional stress intensities, nursing additionally perceived high stress intensities in the organizational and physical areas. Thus, the occupational group of nurses proves to be the most stressed by the COVID-19 pandemic. The findings presented here can be used to derive instructions for future actions.

Die anhaltende COVID-19-Pandemie hat große Auswirkungen auf das deutsche Gesundheitssystem. Während die Mehrheit der Infizierten leichte bis mittelschwere Symptome aufweist, treten bei einigen Patienten schwere Krankheitsverläufe mit „acute respiratory distress syndrome“ (ARDS) auf, die eine intensivmedizinische Versorgung erfordern. Die andauernde COVID-19-Pandemie hat Ärzt:innen und Pflegekräfte auf den Intensivstationen vor enorme emotionale und körperliche Herausforderungen gestellt. Die Auswirkungen dieser Herausforderungen betreffen die körperliche und geistige Leistungsfähigkeit des Gesundheitspersonals. Bislang wurden keine Informationen über teamspezifische Belastungen, mögliche Entlastungsstrategien, positive und negative Erfahrungen sowie Wünsche für eine Verbesserung der Situation erhoben.

Ziel der Untersuchung war die Erhebung von teamspezifischen Faktoren für die Mitarbeitenden einer internistischen Intensivstation in einem Krankenhaus der Maximalversorgung, um damit Handlungsimpulse für zukünftige Krisen zu generieren, die möglicherweise auch auf andere Stationen übertragbar sind.

## Methode

Im Juli 2021 haben Austauschtreffen der pflegerischen und ärztlichen Mitarbeitenden der internistischen Intensivstation der Universitätsklinik Köln stattgefunden. Es handelt sich um eine Intensivstation mit 14 Betten. Bis Juli 2021 wurden insgesamt 191 Patienten und Patientinnen behandelt. Aufgrund der baulichen Begebenheiten wurde die Station im Mischbetrieb gefahren, das heißt, es wurden sowohl Patienten mit einer führenden COVID-19-Infektion als auch Patienten mit anderen internistischen Erkrankungen ohne eine COVID-19-Infektion behandelt. Ein Arzt/Ärztin behandelt im Durchschnitt 7, eine Pflegekraft bis zu 4 Patienten, im Durchschnitt ca. 2,3 Patienten (abhängig von Schicht- und Wochenendbetrieb). Es werden alle gängigen Verfahren der intensivmedizinischen Maximalversorgung im universitären Bereich angeboten. Die Teilnahme an der Evaluation war freiwillig. Ein Ethikantrag wurde nicht gestellt, da keine Beteiligung von Patienten oder Nutzung von Patientendaten stattgefunden hat.

Als Impuls für den inhaltlichen Austausch und die Generierung entsprechenden Datenmaterials wurden drei Fragen zu Belastungsfaktoren, zu positiven Erfahrungen und Entlastungsfaktoren sowie zu Wünschen und Forderungen vorbereitet. Die dazu gehörigen Fragekarten waren für alle Teilnehmenden die gleichen, konnten aber aufgrund einer Kennzeichnung berufsgruppenspezifisch nach Ärzt:innen und Pflegekräften ausgewertet werden. Die Evaluation wurde von einem externen Moderator durchgeführt. Es fand sowohl eine offene Diskussion in der Gesamtgruppe, Einzelarbeit als auch definierte Redeslots für die persönliche Vorstellung statt.

Antworten konnten in Form von Freitext abgegeben werden, zusätzlich sollte auf einer Skala von 1 bis 10 die persönlich empfundene Intensität der benannten Belastung bewertet werden. Schließlich war auf der gleichen Karte zu vermerken, ob es sich bei der Belastung um eine punktuelle, oft wiederkehrende oder dauerhafte Belastung handelte.

Folgende Fragen wurden gestellt:

1. „Was hast Du während der Corona-Pandemie in Deiner Arbeit auf der Intensivstation als belastend empfunden?“
2. „Was war für Dich in dieser Zeit positiv? Hat diese positive Erfahrung zu einer Entlastung geführt (Ja/Nein)? Warum hat diese positive Erfahrung (nicht) zu einer Entlastung geführt?“
3. „Welche Wünsche/Forderungen würdest Du nennen, die aus Deiner Sicht zur Entlastung in der Zeit der Corona-Pandemie hätten beitragen können? Und von wem hättest Du die Erfüllung dieser Wünsche/Forderungen erwartet?“

In einem nächsten Schritt wurden die Teilnehmenden gebeten, die für sie gravierendsten Belastungssituationen in einer Rundenkommunikation zu benennen. Zu einzelnen Punkten gab es Reaktionen der anderen Teilnehmenden, die das Gesagte verstärkten oder durch eigene Erfahrungssituationen ergänzten. An diesen ersten Teil schloss sich eine weitere Runde an, zu der die Teilnehmenden aufgefordert wurden, jeweils eine positive Erfahrung aus den letzten Monaten zu benennen. An manchen Stellen konnte dabei nochmals nachgefragt werden, ob das positiv Erlebte auch als Entlastung erfahren werden konnte. Die gesammelten Daten der Austauschtreffen wurden anschließend in Tabellenform transkribiert und wie folgt ausgewertet.

Für eine erste Typisierung wurden die Freitextantworten zu den Belastungserfahrungen in drei relevante Kategorien gruppiert:

1. Emotional: dieser Kategorie wurden Antworten zugeordnet, die entweder bereits selbst die Beschreibung „emotional belastend“ enthielten (z. B. „Emotionale Belastung durch schwere Krankheitsverläufe, viele Todesfälle, fehlende Behandlungsoptionen“), oder eindeutig emotional belastende Situationen beschrieben („viele Patienten versterben, schwere Krankheitsverläufe oder kein Erfolgserlebnis“). Darüber hinaus wurden der Kategorie Äußerungen zugeordnet, die in einem weiteren Sinne als belastend interpretiert werden konnten (z. B. Notwendigkeit einer Triage, Einschränkung sozialer Kontakte).
2. Körperlich: Aussagen zur körperlichen Belastung zeichnen sich durch eine hohe Eindeutigkeit aus. Durch immer wiederkehrende Signalbegriffe wie „Körperliche Belastung“, „Arbeitsbelastung“, „Tragen der Schutzkleidung über viele Stunden“, „Körperliche Anstrengung“, lassen die Antworten eine eindeutige Anzeige körperlicher Belastung erkennen.
3. Organisatorisch: Eine Rolle spielten hierbei vor allem Herausforderungen in der Kommunikation, die als unterschiedlich empfundenen Behandlungskonzepte verschiedener Oberärzte sowie das Erledigen zusätzlicher Aufgaben außerhalb des Zimmers oder das Arbeiten alleine im Zimmer.

Zur quantitativen Auswertung wurde diese Zuordnung dann mit den entsprechenden Intensitätsangaben nach Berufsgruppen getrennt in eine Datenbank eingetragen. Da die Intensität der Belastungssituation von den Teilnehmenden mit max. 10 angegeben werden konnte, ist dies der höchstmöglichste Belastungswert.

Für die Flächenberechnung im Spinnendiagramm wurde eine trigonometrische Berechnung mit der Formel A = 1/2 × b × c × sin alpha für die Teildreiecke durchgeführt und anschließend die Gesamtsumme durch Addition der Teilergebnisse ermittelt.

## Ergebnisse

### Teilnahme

Insgesamt haben 32 Mitarbeitende teilgenommen, davon 10 Ärzt:innen. Das mediane Durchschnittsalter der Teilnehmenden lag bei 32 (IQR 28–37) (Pflegekräfte) und 39 (IQR 29–46) (Ärzt:innen) Jahren, davon waren 66 % (Pflegekräfte) und 22 % (Ärzt:innen) weiblich.

Es konnte so ein Gesamtdatensatz von 270 Antworten generiert werden, davon 39 Datensätze zu den Belastungssituationen von Ärzt:innen und 101 Datensätze von Pflegekräften. Es gab 13 Datensätze zu positiven Erfahrungen und Entlastungssituationen aufseiten der Ärzt:innen und 57 in der Gruppe der Pflegekräfte. Für die dritte Frage nach Wünschen und Forderungen konnten 17 Datensätze bei Ärzt:innen und 43 bei Pflegenden erhoben werden.

### Quantitative Auswertung

Bei den Ärzt:innen konnten 35 von 39 der abgegebenen Antwortkarten, bei den Pflegekräften 89 von 101 Karten in die quantitative Auswertung einbezogen werden. Alle Freitexteingaben können im Anhang eingesehen werden. Bei den nicht berücksichtigten Karten wurden entweder keine Angaben zur Intensität und/oder zur Belastungsform gemacht. Der Median der angegebenen wahrgenommenen Intensität der Belastung (maximal 10 möglich) lag bei Ärzt:innen bei 7 (IQR 5,25–8,5) bzw. 8 (IQR 7–9) beim Pflegepersonal.

**Figure 1:**
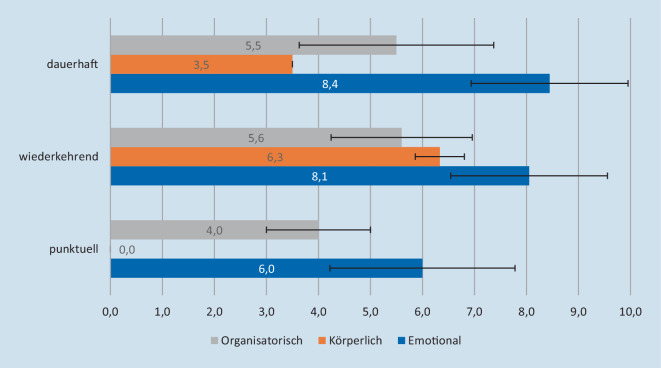

Durch die im Methodenteil beschriebene Vorgehensweise konnten die Aussagen den Kategorien „emotional“, „körperlich“ und „organisatorisch“ zugeordnet und entsprechend mit dem angegebenen Intensitätsfaktor versehen werden. Die Abb. 1 zeigt grafisch die durchschnittlichen Belastungswerte für Ärzt:innen.

Während das Auftreten punktueller und wiederkehrender Belastungsfaktoren in beiden Berufsgruppen ähnlich verteilt ist, zeigt sich ein größerer Anteil dauerhafter Belastungsfaktoren beim Pflegepersonal im Vergleich zu Ärzt:innen.

**Figure 2:**
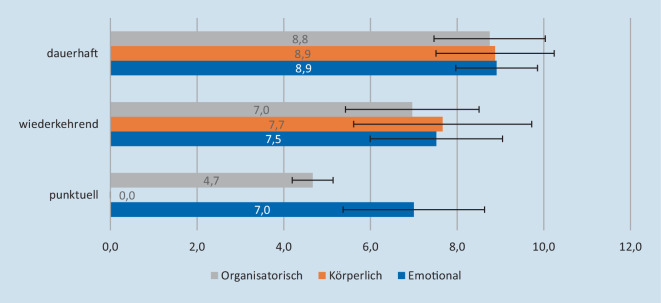

Die Werte der Belastungsfaktoren unterscheiden sich beim Pflegepersonal im Vergleich zu den Ärzt:innen. Die Abb. 2 zeigt die Auswertung des Belastungsfaktoren für das Pflegepersonal.

Die höchsten Werte liegen bei den Pflegekräften bei den dauerhaft empfundenen Belastungsformen, die sowohl emotional, körperlich als auch organisatorisch waren (Mittelwert 9). Auch bei den wiederkehrenden Belastungsformen wurden hohe Werte erhoben.

Punktuell auftretende Belastungsfaktoren waren sowohl bei den Ärzt:innen als auch beim Pflegepersonal eher gering. Körperliche Belastungsformen sind von keiner Berufsgruppe als punktuell wahrgenommen wurden, sondern fast immer als durchgängige Belastungsform für die Zeit der Pandemie.

**Figure 3:**
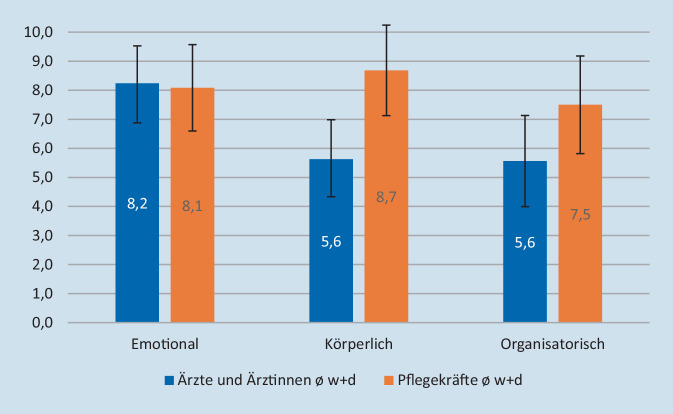

**Figure 4:**
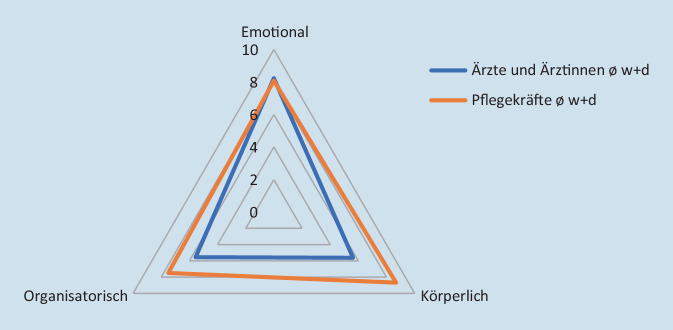

Für den direkten Vergleich der beiden Berufsgruppen haben wir die Intensitätsmessungen für wiederkehrende und dauerhaft wahrgenommene Belastungsformen zusammengefasst und miteinander verglichen (Abb. 3). Diese repräsentieren bei Ärzt:innen 86 % und bei den Pflegekräften 90 % der angegebenen Zahl der Belastungsformen (Abb. 4).

Neben der fast gleichen starken emotionalen Belastung beider Berufsgruppen (Mittelwert 8), werden hier die organisatorische (Mittelwert 8), aber vor allen Dingen auch die körperliche Belastung (Mittelwert 9) im Pflegebereich deutlich. Die Gesamtbelastung im pflegerischen Bereich liegt bei den wiederkehrenden und dauerhaften Belastungsformen bei 85 Flächeneinheiten im Vergleich zu 53 Flächeneinheiten bei den Ärzt:innen.

### Qualitative Auswertung

Unter qualitativen Gesichtspunkten wird deutlich, dass für Pflegekräfte das lange Arbeiten in kompletter Schutzkleidung, insbesondere mit FFP2-Maske, als besonders anstrengend empfunden wurde. Diese Form körperlicher Belastung wurde 8‑mal explizit genannt und aufgrund durchweg hoher Intensitätswerte als in besonderem Maße anstrengend empfunden. Daneben finden sich weitere Anzeigen zur körperlichen Belastung, z. B., dass Pflegemaßnahmen, die ansonsten zu zweit ausgeführt wurden, aufgrund der Isolation alleine durchgeführt werden mussten oder auch die hohe körperliche Anstrengung, welche mit der Lagerung häufig adipöser COVID-19-Patient:innen verbunden war. Weitere, nicht näher differenzierte Anzeigen zur körperlichen Belastung können aufgrund der mündlichen Rückmeldungen in den Austauschtreffen mit all diesen Formen verbunden werden.

Aus pflegerischer Perspektive als emotional belastend empfunden wurde vor allem die Vielzahl der Fälle der an COVID-19 erkrankten Patient:innen, die hohe Sterblichkeitsrate selbst bei relativ jungen Patient:innen sowie das Erleben verzweifelter Patient:innen oder Angehöriger. Als zusätzlich belastend wurde die Sterbebegleitung erlebt, die oftmals aufgrund ebenfalls infizierter, sich in Quarantäne befindender Angehöriger allein von den Pflegekräften (und Ärzt:innen) geleistet werden musste. Die Angst vor einer Eigeninfektion, besonders in der Anfangszeit der Pandemie, war besonders hoch. Es wurde auch mehrfach die Angabe gemacht, Angst vor einer Übertragung auf die eigene Familie zu haben.

Die hohe Sterberate relativ junger Patient:innen wurde ebenfalls von den Ärzt:innen als emotional belastend angezeigt (7-mal genannt). Für die Mediziner:innen, und hier vor allem für die Oberärzte als Hauptentscheidungsträger, waren Formen einer „weichen Triagierung“ belastend. Diese betraf vor allem das mangelnden Kapazitäten geschuldete Ablehnen von Patient:innen aus externen Kliniken, die man bei geringerer Auslastung übernommen hätte.

Herausforderungen, die sich auf organisatorischer Ebene für beide Berufsgruppen ergaben, waren vor allem die Vielzahl der gleichzeitig zu bewältigenden Aufgaben wie häufige Telefonate, Bettenanfragen, Steuerung der Angehörigenbesuche, telefonische Angehörigengespräche, Fahrten zu radiologischen Untersuchungen etc. Als weitere organisatorische Herausforderung wurde gewertet, dass zu Beginn der Pandemie neue Beatmungsgeräte angeschafft und neue Beatmungsmodi eingeführt wurden, ohne eine tiefgehende qualitative Einarbeitung zur Verfügung stellen zu können. Als unterschiedlich empfundene Behandlungskonzepte verschiedener Oberärzte wurden ebenfalls kritisch angemerkt.

### Positive Erfahrungen und Entlastung

Die Rückmeldungen zu positiven Erfahrungen wurden nicht quantitativ ausgewertet, wohl aber konnten Kategorien gebildet werden, die als Typen positiver Erfahrung verstanden werden können. Einer der wichtigsten Faktoren positiver Erfahrung war der Teamzusammenhalt. Allein 34-mal wurde auf die positive Erfahrung gelingender (interprofessioneller) Zusammenarbeit als Entlastungsfaktor verwiesen. Dabei spielen sowohl Aspekte wie Verlässlichkeit und Kooperation, aber auch kollegialer Austausch, Unterstützung durch Helfer:innen, die zusätzlich aus dem Op.-Bereich kamen, Humor, Zusammenhalt und Solidarität professionsübergreifend eine wichtige Rolle. „Der meistgehörte Satz während der Corona-Pandemie auf der Intensivstation“, so eine Kollegin, war die Frage: „Brauchst Du noch Hilfe?“. Solidarität und Kollegialität erweisen sich in emotional belastenden und körperlich anstrengenden Situationen deutlich als motivierende und stabilisierende Faktoren.

Trotz häufig frustrierender Krankheitsverläufe ist der Sinnaspekt ein weiteres Kriterium, das als entlastend wahrgenommen wurde. Das Gefühl, etwas Sinnvolles getan zu haben, in Verbindung mit Erfolgserlebnissen bei Patient:innen konnte trotz hoher Arbeitsbelastung als positive Erfahrung abgespeichert werden.

Die Kategorien Lernen und Wertschätzung wurden als weitere positive Aspekte wahrgenommen, wenn damit auch nicht immer das Gefühl einer Entlastung verbunden war. Dennoch war der Erwerb einer größeren Fachlichkeit eine positive Erfahrung, und mit kleinen Zeichen der Wertschätzung, sowohl im interprofessionellen Team als auch vonseiten der Angehörigen, war das Gefühl verbunden, gesehen zu werden.

### Wünsche und Forderungen

Die Rückmeldungen zu Wünschen und Forderungen weisen ein breites Spektrum von Verbesserungsvorschlägen und verbesserungswürdigen Situationen auf. Dabei bezogen sich auf pflegerischer Seite die meisten Rückmeldungen auf eine Verbesserung des Personalschlüssels und somit auf die Forderung nach mehr fachlich geschultem Personal, hierin eingeschlossen auch die Bitte um Unterstützung von außen, so z. B. von Psycholog:innen und Seelsorger:innen für die Patienten- und Angehörigenbegleitung bzw. in Sterbesituationen. Aber auch bei Ärzt:innen fand sich die Forderung nach mehr Fachpersonal und Anerkennung.

Eine weitere Forderung bezog sich auf die Verbesserung von berufsgruppenübergreifenden Kommunikationsstrukturen. Die Kommunikation solle, so die Rückmeldung, „klarer, häufiger, sachlicher, ergebnisoffener“ und insgesamt „besser“ werden.

Ebenfalls finden sich Rückmeldungen, die so basale Wünsche wie dem nach Bereitstellung von Getränken (Sprudelwasser) oder anderer, kleiner Formen von Wertschätzung artikulieren.

Weitere Rückmeldungen beleuchten den Bereich vielfältiger organisatorischer Fragen. Hier finden sich Forderungen nach einer gemischten Belegung von Intensivbetten (COVID-19-erkrankte Patienten mit anderen internistischen Erkrankungsbildern), Überprüfung alternativer Schichtmodelle, Verbesserung der Zusammenarbeit unterschiedlicher Abteilungen, Bildung von Lagerungsteams, Abbau der Dokumentationsfülle und verbesserte oberärztliche Therapieabsprachen. Schließlich finden sich noch Rückmeldungen, die sich eine Verbesserung der Pausensituation wünschen.

## Diskussion

Ziel dieser Arbeit war es, die Belastungsformen und deren Intensität innerhalb eines Teams in einem Krankenhaus der Maximalversorgung zu evaluieren. Es zeigt sich hier ein erheblicher Unterschied in der wahrgenommenen Belastungsintensität von Pflegekräften und Ärzt:innen innerhalb des Teams. Während beide Berufsgruppen gleich hohe emotionale Belastungsintensitäten wahrnahmen, wurden bei der Pflege zusätzlich fast gleich starke Belastungsintensitäten im organisatorischen und körperlichen Bereich wahrgenommen. Damit erweist sich die Berufsgruppe der Pflegenden als am stärksten durch die COVID-19-Pandemie belastet. Als positiv und entlastend empfunden wurden von beiden Berufsgruppen der Teamzusammenhalt (interprofessionelle Kooperation, Verlässlichkeit, Zusammenhalt, kollegialer Austausch und Humor). Ebenso der Sinnaspekt der Arbeit hatte in den Aussagen eine hohe Bedeutung. Als Wünsche und Forderungen wurden von beiden Berufsgruppen ein verbesserter Personalschlüssel durch fachlich geschultes Personal angegeben. Verbesserung organisatorischer Abläufe würden aus Sicht der Befragten ebenfalls zu einer deutlichen Entlastung führen. Ein wichtiger Punkt sowohl für Ärzt:innen als auch Pflegekräfte ist der Wunsch nach Zeichen von Wertschätzung durch Vertreter:innen der Klinik.

Die weltweite COVID-19-Pandemie hat Auswirkungen auf alle Bereiche der Gesellschaft, insbesondere jedoch auf den medizinischen Bereich. Schon im April 2020 wurde auf die Auswirkungen auf die Mitarbeiter:innen im Gesundheitsdienst und hier besonders auf diejenigen, die direkt mit der Versorgung der COVID-19-Patienten betraut waren, hingewiesen. In der hochrangig veröffentlichten Publikation von Krystal et al. wird auf Stress und die Reaktion der Beschäftigten im Gesundheitswesen im Rahmen der Pandemie aufmerksam gemacht [3]. Hier wird der Begriff der „versteckten Pandemie“ für Mitarbeiter:innen im Gesundheitswesen geprägt. Dieser wurde auch in einem Gastbeitrag im *New England Journal of Medicine* von Dzau et al. aufgenommen [2]. Hier spricht der Autor von einer parallelen Pandemie für die Mitarbeitenden im Gesundheitssystem. Seitdem haben sich international und national eine Vielzahl von Studien, Metaanalysen und Reviews mit den Auswirkungen und Belastungen der COVID-19-Pandemie auf die Mitarbeitenden im Gesundheitssystem beschäftigt [1, 4–11]. Es werden hier zum Teil unterschiedliche Verfahrensweisen und Ziele untersucht. Die Verfahrensweisen sind in allen Studien ausschließlich auf standardisierte Onlinefragebögen ausgelegt. Das Spektrum der Untersuchungsziele umfasst psychischen Stress bzw. Belastung, Angst, Resilienz, Bewältigungsverhalten bis hin zu organisatorischen bzw. Managementproblemen im Umgang mit der Pandemie. Studien im internationalen Kontext zeigen differente Ausgangssituation in den Versorgungsstrukturen des Gesundheitssystems. So liegt z. B. der Anteil der Intensivbetten in Italien bezogen auf die Bevölkerung deutlich unter dem von Deutschland. Damit ist die Belastungssituation für die Mitarbeitenden im jeweiligen Gesundheitssystem sowohl quantitativ als auch qualitativ evident anders. Durch diese unterschiedlichen Versorgungsstrukturen sind die Studien teilweise nur schwer miteinander vergleichbar. Studien aus Deutschland benutzen ausschließlich Online-Surveys für die Erhebung ihrer Ergebnisse [5, 6, 10, 11]. Die teilweise großen Datenmengen zeigen zwar, skaliert durch die unterschiedlichen Testverfahren in den Online-Surveys, einen generalisierten Überblick über die Situation bei Mitarbeiter:innen im deutschen Gesundheitsdienst, geben aber darüber hinaus kaum Möglichkeiten, individuelle Empfindungen bzw. Belastungsintensitäten der Mitarbeiter:innen zu erfassen. Häufig werden auch solche Mitarbeitende in die Untersuchungen mit einbezogen, die kaum oder nur wenig Kontakt mit der Versorgung von COVID-19 Patient:innen hatten. Ferner werden keine teamspezifischen Unterschiede zwischen den Berufsgruppen erfasst, sondern nur Gruppen miteinander verglichen, die unterschiedlichen Teams angehören. Zu vermuten ist, dass aufgrund regional unterschiedlicher Hospitalisierungsraten und hierdurch unterschiedlich stark ausgelasteter Intensivstationen die Belastungen in den jeweiligen Teams sehr unterschiedlich sind. In der hier vorliegenden Studie wurden durch eine direkte Teambefragung und die Möglichkeit individueller Beschreibung von Belastungssituationen teamspezifische Informationen gewonnen, die sowohl quantitativ als auch qualitativ ausgewertet werden konnten. Eine ebenfalls immer wieder auftauchende Sorge oder Angst ist, sich selbst anzustecken und damit auch seine Angehörigen zu gefährden. Dies wird nicht nur bei unserer Befragung deutlich, sondern auch in schon publizierten Studien[6]. Allerdings ändert sich die Sorge um die eigene Infektion im Verlauf der Pandemie. Während im vergangenen Jahr vor allem das Risiko für sich selbst und Angehörige durch eine potenzielle Ansteckung genannt wurde, traten seit Einführung der Impfung andere Sorgen in den Vordergrund.

Im Gegensatz zu den bisher durchgeführten Studien konnten wir in unserer Studie durch die hier angewendete Auswertungsanalytik eine hohe Intensität in körperlichen Belastungswerten für das Pflegepersonal detektieren. Das Tragen von persönlicher Schutzausrüstung über einen langen Zeitraum, Pflegemaßnahmen, die alleine durchgeführt werden mussten, sowie die körperlich anstrengende Versorgung häufig adipöser COVID-Patient:innen verbunden mit deren medizinisch notwendigen Lagerungswechseln waren immer wiederkehrende Kommentare im Austauschgespräch. Aber auch die hohe emotionale Belastung sowohl bei Ärztinnen und Ärzte als auch beim Pflegepersonal fielen auf, wenn auch mit teils unterschiedlichen Schwerpunkten. Während bei beiden Berufsgruppen die hohe Sterblichkeit selbst bei jungen Patient:innen als emotional belastend empfunden wurde, war es bei Ärzt:innen zusätzlich vor allem die Kommunikation mit Angehörigen, bei Pflegekräften die eigene Isolation im Zimmer, aber auch die der Patientin/des Patienten und ihrer/seiner Angehörigen. Im Gegensatz zu großen Online-Surveys gab es in unserer Studie die Möglichkeit, sowohl positive Erfahrungen und Entlastungsmomente als auch Wünsche und Forderungen für die Zukunft zu eruieren. Durch die Methode, hier eine gesamte Teamabfrage von Ärzt:innen und Pflegekräften durchzuführen, konnte die besondere Bedeutung des Teamgedankens herausgearbeitet werden. Wörter wie Teamzusammenhalt, Verlässlichkeit, Kooperation, Solidarität und Wertschätzung wurden in beiden Berufsgruppen häufig genannt. Diese Attribute stellen wichtige positive Erfahrungen und Entlastungsmomente für das interprofessionelle Arbeiten dar. Die Ergebnisse der Abfrage von Wünschen und Forderungen für den weiteren Verlauf zeigen hier vier wesentliche Punkte: Verbesserung der Wertschätzung, Personalzusatz, bessere Kommunikation und Einsatz von Psycholog:innen und Seelsorger:innen, vor allem am Lebensende. Insbesondere Letzteres wird auch in vielen nationalen und internationalen Studien als Schlussfolgerung angegeben [10].

Die hier durchgeführte Untersuchung weist allerdings auch einige Nachteile auf. Durch den monozentrischen Charakter und die geringe Teilnehmerzahl können leicht Verschiebungen in der Interpretation stattfinden. Wir können nicht für alle Antworten ausschließen, dass diese bisweilen einer anderen Kategorie (emotional, organisatorisch und körperlich) zugeordnet wurden, als vom Teilnehmenden mit der Aussage intendiert. Mit den oben aufgeführten Argumenten haben wir keine klassischen Messskalen für die Erhebung eingesetzt. Das macht einen Vergleich mit anderen Studien schwieriger und damit statistisch schlechter auswertbar. Auch die Zuordnung des Terminus Arbeitsbelastung zum Oberbegriff „körperlich“ ist nicht immer klar von allen Aussagen möglich, da psychische und emotionale Faktoren dieses Empfinden wesentlich beeinflussen können. Wir haben uns bemüht, die Zuordnung möglichst einheitlich durchzuführen.

Durch die hier herausgearbeiteten Erkenntnisse können für die Zukunft konkrete Handlungsanweisungen abgeleitet werden. Die hohe körperliche Belastung macht einen zusätzlichen Personaleinsatz notwendig. Das dies in der aktuellen angespannten Personalsituation für Pflegekräfte kaum umzusetzen ist, können zumindest medizinisch-technische Angestellte o. Ä. für nicht ärztliche und pflegerische Aufgaben eingesetzt werden. Ebenso ist die Wertschätzung der Mitarbeiter wichtig. Dies kann durch persönliche Gespräche der erweiterten Klinikleitung, aber auch durch Bereitstellung, z. B. von Getränken, oder Ähnliches zum Ausdruck gebracht werden. Emotionale Belastungen können durch zusätzliche Teams von Psychologen/Seelsorgern zu einer Entlastung führen. Organisatorisch würden Teambesprechungen und verbesserte Absprachen unnötige und zeitaufwendige Managementprobleme vermeiden.

Zusammenfassend kann aus unseren Untersuchungsergebnissen eine Reihe von Ansatzpunkten für eine zukünftige Verbesserung von Belastungsintensitäten bzw. Belastungsformen von Pflegekräften und Ärzt:innen innerhalb eines Behandlungsteams eruiert werden. Sowohl emotionale Belastungsformen in beiden Berufsgruppen als auch körperliche und organisatorische Belastungsformen in der Gruppe der Pflegenden sind mit einer hohen Intensität belegt. Gerade die Berufsgruppe der Pflegenden gilt als die am stärksten in der Corona-Pandemie belastete Gruppe.

## References

[1] Bohlken J, Schömig F, Lemke MR (2020). COVID-19 pandemic: stress experience of healthcare workers. Psychiat Prax.

[2] Dzau VJ, Kirch D, Nasca T (2020). Preventing a parallel pandemic—A national strategy to protect clinicians’ well-being. N Engl J Med.

[3] Krystal JH, McNeil RL (2020). Responding to the hidden pandemic for healthcare workers: stress. Nat Med.

[4] Kunzler AM, Röthke N, Günthner L (2021). Mental burden and its risk and protective factors during the early phase of the SARS-CoV-2 pandemic: systematic review and meta-analyses. Glob Health.

[5] Morawa E, Schug C, Geiser F (2021). Psychosocial burden and working conditions during the COVID-19 pandemic in Germany: The VOICE survey among 3678 health care workers in hospitals. J Psychosom Res.

[6] Paffenholz P, Peine A, Hellmich M (2020). Perception of the 2020 SARS-CoV-2 pandemic among medical professionals in Germany: results from a nationwide online survey. Emerg Microbes Infec.

[7] Sanghera J, Pattani N, Hashmi Y (2020). The impact of SARS-coV-2 on the mental health of healthcare workers in a hospital setting—A systematic review. J Occup Health.

[8] Schulze S, Holmberg C (2021). Importance of and burden on nursing staff during the corona crisis. Public Heal Forum.

[9] Shechter A, Diaz F, Moise N (2020). Psychological distress, coping behaviors, and preferences for support among New York healthcare workers during the COVID-19 pandemic. Gen Hosp Psychiat.

[10] Skoda E-M, Teufel M, Stang A (2020). Psychological burden of healthcare professionals in Germany during the acute phase of the COVID-19 pandemic: differences and similarities in the international context. J Public Health.

[11] Zerbini G, Ebigbo A, Reicherts P et al (2020) Psychosocial burden of healthcare professionals in times of COVID-19—a survey conducted at the University Hospital Augsburg. Gms Ger Med Sci 18:Doc5. 10.3205/000281 10.3205/000281 PMC7314868 32595421

